# Novel Regulators of Macropinocytosis-Dependent Growth Revealed by Informer Set Library Screening in Pancreatic Cancer Cells

**DOI:** 10.3390/metabo12090831

**Published:** 2022-09-02

**Authors:** Sang Hoon Kim, Jae Ho Song, Min Ji Kim, Mun Gu Song, Angel A. Ku, Sourav Bandyopadhyay, Frank McCormick, Sung Eun Kim

**Affiliations:** 1 Department of Biosystems and Biomedical Sciences, College of Health Sciences, Korea University, Seoul 02841, Korea; 2 Department of Integrated Biomedical and Life Sciences, College of Health Sciences, Korea University, Seoul 02841, Korea; 3 Helen Diller Comprehensive Cancer Center, University of California, San Francisco, CA 94158, USA; 4 Bioengineering and Therapeutic Sciences, University of California, San Francisco, CA 94158, USA; 5 National Cancer Institute RAS Initiative, Cancer Research Technology Program, Frederick National Laboratory for Cancer Research, Frederick, MD 21701, USA

**Keywords:** macropinocytosis, pancreatic cancer, Informer set library screening, cancer metabolism, nutrient scavenging

## Abstract

Cancer cells utilize multiple nutrient scavenging mechanisms to support growth and survival in nutrient-poor, hypoxic tumor microenvironments. Among these mechanisms, macropinocytosis has emerged as an important pathway of extracellular nutrient acquisition in cancer cells, particularly in tumors with activated RAS signaling, such as pancreatic cancer. However, the absence of a clinically available inhibitor, as well as the gap of knowledge in macropinocytosis regulation, remain a hurdle for its use for cancer therapy. Here, we use the Informer set library to identify novel regulators of macropinocytosis-dependent growth in pancreatic cancer cells. Understanding how these regulators function will allow us to provide novel opportunities for therapeutic intervention.

## 1. Introduction

Tumors rewire metabolic pathways to meet the energetic and redox demands needed to build biomass and to survive and proliferate within nutrient-deprived tumor microenvironments [1]. Cancer cells can upregulate scavenging pathways, such as autophagy or macropinocytosis, which function to provide cells with nutrients that support growth. In particular, the breakdown of extracellular protein through macropinocytosis can contribute to the supply of nutrients in tumors, and high levels of macropinocytosis have been observed in human tumors [2]. In particular, studies have shown that KRAS mutant cancer cells, including pancreatic ductal adenocarcinoma (PDAC), upregulate macropinocytosis to import extracellular protein to support growth upon nutrient depletion [3]. Similarly, high levels of macropinocytic uptake are observed not only in PDAC murine models, but also in human PDAC specimens [2,4]. In addition, in a different perspective, macropinocytic uptake can influence drug efficacy and has been considered to be the underlying reason for the success of nab-paclitaxel, the FDA-approved nanoparticle albumin-bound form of paclitaxel [5]. The metabolic contributions of macropinocytosis and the signaling pathways that regulate it remain poorly understood and, thus, understanding these regulations will be important to reveal potential therapeutic targets and enhance drug efficacy.

Although it is well established that the internalization of extracellular proteins through macropinocytosis is utilized by cancer cells, no clinically useful inhibitors are available to regulate macropinocytosis in pathological processes. Currently, the Na^+^/H^+^ exchanger (NHE1) inhibitor 5-(N-ethyl-N-isopropyl)-amiloride (EIPA) and the epithelial sodium channel inhibitor dimethyl amiloride are the most effective and selective pharmacological tools used to inhibit macropinocytosis. However, possible effects of these inhibitors on ion transport, intracellular pH, and the cytoskeleton independent of macropinocytosis limit their use as pharmacological inhibitors. Therefore, the discovery of novel compounds that regulate macropinocytosis is important for the development of pharmacological macropinocytosis regulators.

Here, we used the Informer set library of compounds, consisting of FDA-approved drugs and clinical candidates, to screen for positive and negative regulators of macropinocytosis using culture conditions of glutamine deprivation supplemented with extracellular protein. The ability of cancer cells to survive and grow in these conditions is dependent on macropinocytosis. From this screen, we identified groups of compounds that lead to an increase or decrease in macropinocytosis-dependent growth. We find that, among these compounds, several overlap in their targets, suggesting that these targets function as macropinocytosis regulators. Several targets have been previously identified to regulate macropinocytosis, whereas others are novel regulators of macropinocytosis identified in this study. Further examination to determine the mechanism of these regulators in the uptake, processing, or utilization of extracellular protein for macropinocytosis may unravel potential uses for cancer therapeutics.

## 2. Materials and Methods

### 2.1. Cell culture and Reagents

Cells were grown in DMEM (11965-092, Thermo Fisher Scientific, Waltham, MA, USA) with 10% FBS (F2442, Sigma-Aldrich, St. Louis, MO, USA). Glutamine starvation medium was made using DMEM with no glutamine (11960-044, Thermo Fisher Scientific) with 10% dialyzed FBS (26400-044, Thermo Fisher Scientific). When indicated, 5% bovine serum albumin (BSA) (A1470, Sigma-Aldrich) or 4 mM glutamine (25030-081, Thermo Fisher Scientific) was added to the glutamine starvation medium. When indicated, cells were treated with Torin1 (4247, Tocris Bioscience, Bristol, UK) or EIPA (1154-25-2, Tocris).

### 2.2. Dextran Uptake Assay

For imaging of the TMR–Dextran uptake, cells were plated on glass-bottom dishes (P06G-1.5-20-F, MatTek, Ashland, MA, USA) and incubated in 0.5 mg/mL TMR–Dextran (S11368; Molecular Probes, Eugene, OR, USA) for 2 h to allow macropinocytosis to proceed. Cells were then washed twice with PBS, and the medium was replaced with complete medium without TMR–Dextran in order to image using confocal microscopy.

### 2.3. Cell Viability Assay

Cell viability was measured using the CellTiter-Glo™ luminescent cell viability assay (G7570, Promega, Madison, WI, USA) according to the manufacturer’s instructions. Briefly, cells were plated in duplicate onto 96-well plates in complete medium and, the next day, medium was exchanged into glutamine starvation medium with or without 5% BSA or 4 mM glutamine. When indicated, Torin1 or EIPA was added to the medium at various concentrations. After 3 days, cells were incubated for 15 min with CellTiter-Glo™ reagent and luminescence was measured using a 96-well plate reader (GloMax Discover Microplate Reader, Promega).

### 2.4. Generation of NHE1 Knockdown Cells

A sequence for targeting NHE1 mRNA was designed by using splashRNA [6] and cloned into LT3GEPIR [7]. The antisense guide sequence for NHE1 mRNA used is TTAGAAACTAAGATTGGTCTGA. Lentiviral vector expressing shRNA was transfected into HEK293T cells along with lentiviral packaging plasmids (pMD2.G and psPAX2) using lipofectamine 2000 (11668-019, Thermo Fisher Scientific). Then, 48 h after transfection, supernatant with virus particles was collected, centrifuged, filtered (0.45 μm), and stored at 4 °C for immediate use. Then, MiaPaCa2 parental cells plated in 6-well plates (1 × 10^5^ cells per well) were infected in media containing virus and 10 μg/mL of polybrene. After 24 h of infection, selection of transduced cells was performed using 2 μg/mL puromycin as selection marker.

### 2.5. Quantitative Real-Time PCR

The RNA was extracted from cells using RiboEX (301-001, GeneAll, Seoul, Republic of Korea) according to the manufacturer’s instructions, and 3 μg of total RNA was used for reverse transcription using the reverse transcriptase (EP0441, Thermo Fisher Scientific) and dNTP mix (R0192, Thermo Fisher Scientific). For qPCR, cDNA was amplified using SYBR green PCR master mix (A25780, Applied Biosystems, Foster City, CA, USA) following regular qPCR protocol for 45 cycles on a Quantstudio 7 Flex Real-Time PCR (Applied Biosystems). Target gene copy numbers were compared to the copy number of β-actin. Primer sequences used for NHE1 mRNA are forward, as follows: 5′-GAACTGGACCTTCGTCATCAGC-3′; in reverse, they are as follows: 5′-GGTCAGCTTCACGATACGGAAC-3′.

### 2.6. Informer Set Library Screening

For the Informer set library screening, cells were plated in 40 μL of medium per well in glutamine starvation medium supplemented with 5% BSA. The next day, intermediate stock concentrations of 1, 5, 25, and 125 μM of the Informer set library compounds were added at 10 μL, resulting in final concentrations of 0.2, 1, 5, and 5 μM. Additionally, intermediate stock concentrations of 0.2, 1, 5, and 25 μM of Torin1 and 4, 20, 100, and 500 μM of EIPA were added at 10 μL, resulting in final concentrations of 0.04, 0.2, 1, and 5 μM of Torin1, and 0.8, 4, 20, and 100 μM of EIPA. Cell viability was measured after 3 days using the CellTiter-Glo™ luminescent cell viability assay, and 4 replicates were used to calculate the average area under the curve (AUC) for each compound.

### 2.7. Statistics

The indicated p values were obtained using Student’s t test (**, *p* < 0.01, *, *p* < 0.05).

## 3. Results

### 3.1. PDAC Cells Utilize Extracellular Protein to Enhance Survival in Glutamine-Deprived Conditions

**Figure 1:**
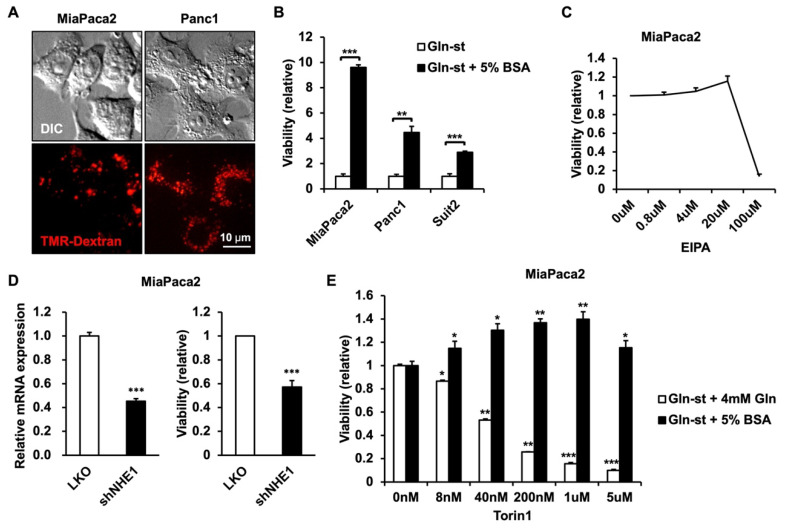
PDAC cells utilize extracellular protein to enhance survival in glutamine-deprived conditions. (**A**) Representative images of MiaPaca2 and Panc1 cells incubated with 70 kDa TMR–Dextran for 2 h, demonstrating macropinocytic uptake. (**B**) Relative viability of MiaPaca2, Panc1, and Suit2 showing the fold increase in viability induced by the supplementation of 5% BSA compared to glutamine starvation conditions for 3 days. The indicated *p*-values were obtained using Student’s t test (***, *p* < 0.001, **; *p* < 0.01). (**C**) Relative viability of MiaPaca2 cells cultured in glutamine starvation conditions supplemented with 5% BSA and treated with various concentrations of EIPA for 3 days. (**D**) Relative expression of NHE1 mRNA and corresponding viability of NHE1-depleted MiaPaca2 cells cultured in glutamine starvation conditions and supplemented with 5% BSA. (**E**) Relative viability of MiaPaca2 cells cultured in glutamine starvation conditions supplemented with either 4 mM glutamine or 5% BSA and treated with various concentration of Torin1 for 3 days. Data in B, C, E are presented as mean +/− SEM, *n* = 3; data in D are presented as mean +/− SEM, *n* = 4. The indicated *p*-values were obtained using Student’s t test (*, *p* < 0.05, **, *p* < 0.01, ***, *p* < 0.001).

Glutamine is often depleted in tumor microenvironments, and macropinocytosis serves as a survival mechanism of extracellular nutrient uptake for cancer cells to survive in these conditions [3]. The role of macropinocytosis has been especially well-studied in PDAC cells, and we first sought to identify cells which can utilize macropinocytosis to efficiently survive in glutamine-deprived conditions. The PDAC cells have been previously described to undergo macropinocytosis, and we confirmed the uptake of 70 kDa tetramethylrhodamine (TMR)–dextran, which is a marker for macropinocytic activity, in MiaPaca2 and Panc1 cells (Figure 1A). Survival in glutamine-deprived conditions with or without the supplement of 5% BSA, which is similar to the physiological concentration of albumin [8], was compared in MiaPaca2, Panc1, and Suit2 cells (Figure 1B). Although all three cell lines were able to utilize BSA to enhance survival in glutamine-deprived conditions, MiaPaca2 cells were able to increase survival 9.6-fold with the presence of BSA in these conditions, compared to 4.5-fold and 2.9-fold for Panc1 and Suit2 cells, respectively, suggesting that MiaPaca2 cells can most efficiently utilize BSA in glutamine-deprived conditions and are a suitable model for the screening of regulators of macropinocytosis.

To test whether the currently known macropinocytosis-regulating compounds work as expected in the MiaPaca2 macropinocytosis model, we tested EIPA, a macropinocytosis inhibitor, and Torin1, the mechanistic target of the rapamycin (mTOR) inhibitor, which enhances macropinocytosis-dependent growth [9]. Our results were consistent with previous studies in that EIPA inhibits macropinocytosis-dependent growth between the concentrations of 20 and 100 μM (Figure 1C). Indeed, EIPA is a known inhibitor of Na^+^/H^+^ exchanger (NHE1), and we confirmed that genetic depletion of NHE1 also inhibits macropinocytosis-dependent growth (Figure 1D). Additionally, Torin1 treatment enhances macropinocytosis-dependent growth at concentrations between 8 nM and 1 μM, whereas it inhibits growth of macropinocytosis-independent growth in complete medium (4 mM glutamine supplemented in glutamine-deprived conditions) (Figure 1E). According to previous studies, this can be accounted for by the inhibition of lysosomal activity by mTOR and the relieving of this effect through Torin1 treatment in macropinocytosis-dependent conditions [9]. At high concentrations of 5 μM, it is most likely that Torin1 exhibits growth-inhibiting effects regardless of culture conditions. Overall, these data demonstrate that the use of glutamine-deprived conditions supplemented with 5% BSA in MiaPaca2 cells can be used as a system to screen for macropinocytosis regulators.

### 3.2. Macropinocytosis Regulator Screen using the Informer Set Library

Previous reports of macropinocytosis screens have mostly focused on the initial step of uptake in macropinocytosis, using a 70 kDa dextran uptake as a marker [10]. These results have revealed pathways that lead to the formation of macropinosomes or pathways that block this process [11,12,13]. Although these findings are meaningful, not much is known about the downstream pathways that lead to the utilization of macropinocytosis to increase the survival and proliferation of cancer cells. Therefore, we instead focused on macropinocytosis-dependent cancer cell growth as a unique read-out for the screening process in this study. Through these methods, this screen enabled us to uncover targets that function in various steps of the process in which macropinocytosis of extracellular protein leads to increased growth of cancer cells.

**Figure 2:**
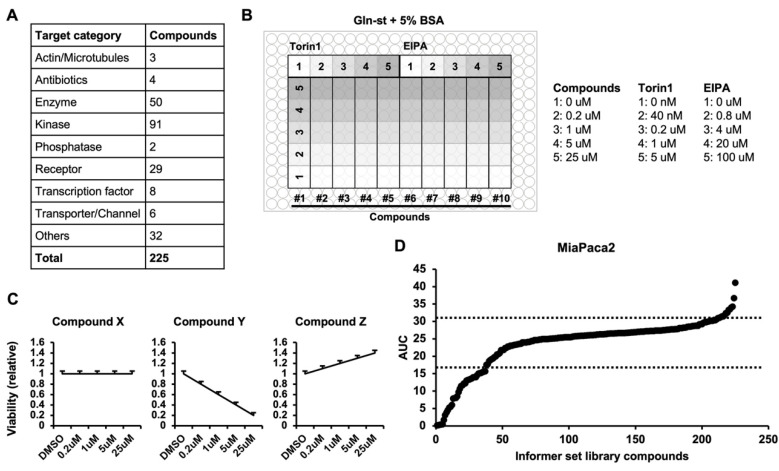
Macropinocytosis regulator screen using the Informer set library. (**A**) Categories of known targets for the 225 compounds composing the Informer set library. (**B**) Plate settings used in the screen demonstrating the treatment concentrations of library compounds, Torin1, or EIPA used in the screening process. (**C**) Examples of AUC calculated from the relative viability of cells treated with individual compounds indicating no change (Compound X), decrease (Compound Y), or increase (Compound Z) in viability. (**D**) Alignment of AUC value of all 225 compounds with the cut-off for positive regulators and negative regulators.

The Informer set library used in our study consists of 225 compounds that target various cancer-related targets (Figure 2A). Our screen was proceeded for a total of 5 days and each compound was treated at various concentrations, ranging from 0.2 to 25 μM, to provide additional information of drug sensitivity (Figure 2B). Furthermore, EIPA and Torin1 were included in each plate to ensure that the macropinocytosis-dependent conditions were consistent. At the end of the screen, the AUC was calculated for each compound from the graph created from the relative viability of compound treatment concentrations normalized to the DMSO control (Figure 2C). If the compound did not have any effect on viability up to 25 μM, the AUC would be 25. An AUC lower than 25 indicates an inhibitory effect, whereas an AUC higher than 25 indicates an enhancement of cell viability, respectively, in these conditions. The mean AUC of all 225 compounds was 23.7, and the bulk of the compounds ranged within the standard deviation, whereas 37 compounds led to a significant AUC decrease (lower than 16.4), and 13 compounds led to a significant AUC increase (higher than 31.0) (Figure 2D).

### 3.3. Positive Regulators of Macropinocytosis-Dependent Growth

**Figure 3:**
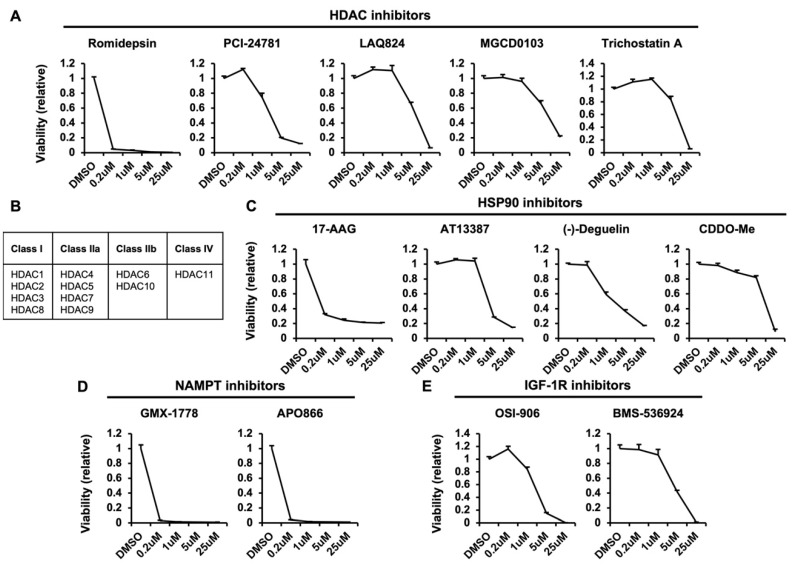
Positive regulators of macropinocytosis-dependent growth. (**A**) Relative viability of cells treated with various concentrations of the HDAC inhibitors romidepsin, PCI-24781, LAQ824, MGCD0103, and trichostatin A. (**B**) Box indicating the distinct classes of the HDAC family and their constituent members. (**C**) Relative viability of cells treated with various concentrations of the HSP90 inhibitors 17-AAG, AT13387, (-)-Deguelin, and CDDO-Me. (**D**) Relative viability of cells treated with various concentrations of the NAMPT inhibitors GMX-1778 and APO866. (**E**) Relative viability of cells treated with various concentrations of the IGF-1R inhibitors OSI-906 and BMX-536924.

The list of 37 compounds that significantly decreased the AUC are shown in Supplemental Table S1. Several compounds overlapped in their targets, which suggests that these targets function as positive regulators of macropinocytosis. Firstly, all five inhibitors of histone deacetylases (HDACs) that exist in the library led to a decrease in AUC in macropinocytosis-dependent conditions; these are romidepsin, PCI-24781, LAQ824, MGCD0103, and trichostatin A (Figure 3A). It was reported that HDAC overexpression in cancer cells is associated with increased macropinocytosis and cellular migration as well as metastatic potential [14]. Specifically, HDAC6 underwent translocation to actin-enriched membrane ruffles in response to growth factor stimulation and became associated with macropinosomes. Additionally, HDAC6-deficient cells were impaired in membrane ruffle formation, macropinocytosis, and migration, leading to a reduction in metastatic capacity. In another study, authors took advantage of this HDAC-associated induction of macropinocytosis, linking the HDAC inhibitor cinnamic acid to neutral red, a molecule which enters cells through macropinocytosis [15]. This study demonstrated an alternative pathway for drug distribution to cancer cells by exploiting HDAC-induced macropinocytosis. The HDACs can be divided into different classes depending on their homology [16], and HDAC6 is a member of the class IIb, which can be inhibited by LAQ824 and trichostatin A (Figure 3B). However, romidepsin, PCI-24781, and MGCD0103 are class I HDAC inhibitors, suggesting that additional HDAC members may also regulate macropinocytosis-dependent growth.

Heat shock protein 90 (HSP90) was another target of compounds that overlapped in the AUC decrease list. Similar to HDACs, all four inhibitors that target HSP90 led to a decrease in AUC; these are 17-AAG, AT13387, (-)-Deguelin, and CDDO-Me (Figure 3C). Indeed, HSP90 was shown to be a prominent substrate of HDAC6 in the study mentioned above, suggesting that it may act through the same mechanism as HDAC6 [14]. As with HDAC6, HSP90 was also recruited to membrane ruffles and macropinosomes, and treatment with the HSP90 inhibitor geldanamycin suppressed membrane ruffling and cell migration. These data suggest that the HSP90 inhibitors in our screen may elicit similar effects on cells to block macropinocytic uptake.

The nicotinamide phosphoribosyltransferase (NAMPT) inhibitors GMX-1778 and APO866 (Figure 3D) and insulin-like growth factor 1 receptor (IGF-1R) inhibitors OSI-906 and BMS-536924 also led to a decrease in AUC (Figure 3E). Here, NAMPT is the rate-limiting enzyme in the nicotinamide adenine dinucleotide (NAD^+^) salvage pathway. The inhibition of NAMPT impairs pancreatic cancer growth in vitro and in vivo [17], and this impairment was rescued by supplementation of nicotinamide mononucleotides [18]. Unlike the two targets mentioned above, HDAC6 and HSP90, it is unlikely that NAMPT functions to upregulate macropinocytic uptake, as it is not known to be localized to the plasma membrane or play a role in membrane trafficking. Instead, it is possible that the catabolism of BSA through macropinocytosis leads to elevated NAD^+^ levels or enhanced NAMPT activity that is important for macropinocytosis-dependent cell growth. In the case of IGF-1R, the underlying mechanism may be related to the observation that IGF-1R activates the RAS/RAF/MAPK signaling pathway [19]. Activation of the RAS pathway is one of the most well-known signaling inputs to enhance macropinocytic uptake [20]. Therefore, IGF-1R activation may support macropinocytosis-dependent growth of pancreatic cancer cells through the activation of the RAS/RAF/MAPK pathway.

### 3.4. Negative Regulators of Macropinocytosis-Dependent Growth

**Figure 4:**
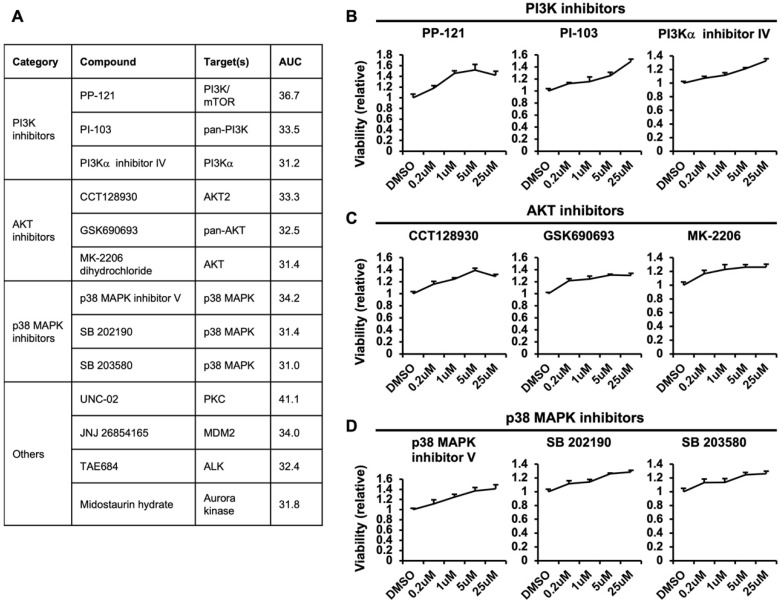
Negative regulators of macropinocytosis-dependent growth. (**A**) Compounds that lead to an increase in AUC, including known targets and AUC value. (**B**) Relative viability of cells treated with various concentrations of the PI3K inhibitors PP-121, PI-103, and PI3Kα inhibitor IV. (**C**) Relative viability of cells treated with various concentrations of the AKT inhibitors CCT128930, GSK690693, and MK-2206. (**D**) Relative viability of cells treated with various concentrations of the p38 MAPK inhibitors p38 MAPK inhibitor V, SB 202190, and SB 203580.

The list of compounds that lead to an increase in AUC and, therefore, macropinocytosis-dependent growth is shown in Figure 4A and Supplemental Table S1. Firstly, there were multiple inhibitors of PI3K or AKT, which are upstream inhibitors of mTOR. As mentioned above, mTOR suppresses the utilization of extracellular proteins through macropinocytosis by inhibiting lysosomal catabolism [9]. Therefore, inhibitors of PI3K or AKT may elicit similar effects on macropinocytosis-dependent growth by functioning as mTOR inhibitors. The PI3K inhibitors PP-121, PI-103, and PI3Kα inhibitor IV (Figure 4B) and AKT inhibitors CCT128930, GSK690693, and MK-2206 dihydrochloride (Figure 4C) were also shown to lead to an increase in AUC. Among these inhibitors, PP-121 and CCT128930 show a similar trend to Torin1 (Figure 1D), in which cell viability is enhanced with increasing concentrations but decreases at a high concentration. These data suggest that in addition to the known phenotypes caused by PI3K and AKT inhibitors, these inhibitors may enhance macropinocytosis-dependent growth of cancer cells, which is an important factor to consider when treating cancers.

Another negative regulator found in our screen was p38 MAPK. The p38 MAPK inhibitors p38 MAPK inhibitor V, SB202190, and SB203580 were found to lead to an increase in AUC (Figure 4D). A study showed that SB202190 inhibits human monocyte-derived dendritic cell uptake of FITC-dextran suggesting the inhibition of macropinocytosis [21], in contrast to our result. However, 10 kDa FITC–dextran was used in this study, which can be taken up by other endocytic mechanisms and, therefore, it is difficult to conclude that this effect is specifically caused through the inhibition of macropinocytosis. In another study, SB202190, but not other inhibitors, such as SB203580, promoted the translocation of transcription factor EB and transcription factor E3 into the nucleus and enhanced autophagy and lysosomal biogenesis independent of p38 MAPK [22]. However, our results demonstrate similar effects of multiple p38 MAPK inhibitors, suggesting that the effect on macropinocytosis-dependent growth is through the function of the p38 MAPK pathway (Figure 4D). Interestingly, a recently discovered downstream mechanism of p38 MAPK is autophagy inhibition through UNC51-like kinase 1 phosphorylation, similarly to mTOR [23]. Although the effects of mTOR on inhibiting macropinocytosis-dependent growth is thought to be through lysosome inhibition more so than autophagy, it will be interesting to investigate whether p38 MAPK also regulates lysosome function or acts through an independent parallel pathway.

## 4. Discussion

Macropinocytosis is a cellular process that has been observed for almost 100 years, and it is known to occur in various cell types [24]. The interest in macropinocytosis from the cancer perspective was sparked by a study showing that mutant RAS pancreatic cancers can utilize macropinocytosis to survive in nutrient-deprived conditions [3]. Several other studies further showed that human pancreatic cancer tissues undergo macropinocytosis and that various substrates can enable cancer cell survival through macropinocytosis [8]. In addition to the importance of macropinocytosis as a nutrient scavenging process for cancer cells, it can also potentially be utilized as a method for drug delivery. Cytosolic antibody delivery could be efficiently achieved by peptides through macropinocytosis induction, which leads to efficient cytosolic delivery [25]. Although these and other recent studies have exemplified the importance of macropinocytosis in cancer cells, there remains a gap in the understanding of macropinocytosis regulation in cancer, as well as an absence of inhibitors that can be used for clinical practice.

Previous studies have sought to use screens to uncover macropinocytosis regulators. Most of these screens focused on the initial step of macropinocytosis by using dextran uptake as an indicator of macropinocytosis. In one study, the uptake of lysine-fixable 70 kDa TMR–dextran of HeLa cells expressing oncogenic HRAS G12D was used in a genome-wide siRNA screen to identify genes involved in controlling mutant HRAS-dependent macropinocytosis [11]. In another study, a systemic screen with a FDA-approved drug library was used to identify compounds that inhibit the uptake of 70 kDa FITC–dextran [12]. Lastly, a genome-wide gain-of-function shRNA screen using Bacillus Calmette-Guerin (BCG) uptake as an indicator of macropinocytosis was performed, and it identified multiple negative regulators of the Wnt pathway as hits [13]. These results identified the Wnt pathway as a strong driver of macropinocytosis, which has been confirmed by several following studies [26,27]. These findings are meaningful and have revealed novel regulators of the major signaling pathways that lead to macropinosome formation. However, they still leave many unanswered questions concerning the regulation of macropinocytosis-dependent growth of cancer cells.

Therefore, in our study, we focused on the growth of cancer cells in conditions that force them to be dependent on macropinocytosis as a read-out for our screen. This way, not only could we identify regulators of macropinocytic uptake in as the previous studies, but we could also identify the regulators of other processes that are needed for the growth of cancer cells in these conditions. Among the positive regulators of macropinocytosis-dependent growth identified in our study, HDAC and its substrate HSP90 were found to localize to membrane ruffles and enhance macropinosome formation [14]. Similarly, IGF-1R was also identified as a positive regulator of macropinocytosis-dependent growth which is most likely through the activation of the RAS pathway [19]. There are multiple HDAC, HSP90, or IGF-1R inhibitors that are in clinical use or trials and whether these compounds can regulate macropinocytosis-dependent growth in these settings is an important question for future studies [28,29,30]. In comparison, NAMPT was also identified as a positive regulator of macropinocytosis-dependent growth in the present study, but it is unlikely that it directly functions to enhance macropinocytic uptake. Instead, it is possible that macropinocytic processing of extracellular protein leads to elevated NAD^+^ levels or enhanced NAMPT activity that is important for macropinocytosis-dependent growth. Further studies will be needed to address these important questions.

Another distinction from previous macropinocytosis screening studies is that, whereas these studies only focused on inhibitors of macropinocytosis, we have also identified compounds that enhance cell growth in macropinocytosis-dependent conditions. As mentioned previously, mTOR inhibitors enhance proliferation in macropinocytosis-dependent culture conditions [9], and we confirmed that PI3K or AKT inhibitors have similar effects, most likely by functioning through mTOR. These results have important implications for the use of PI3K and AKT inhibitors, as they may have unwanted effects of enhancing growth in settings where cells are dependent on extracellular protein utilization. Therefore, these results should be considered when evaluating the efficacy of these inhibitors in the clinic. Another group of compounds that were found to enhance growth of cells in these conditions were p38 MAPK inhibitors, suggesting that p38 MAPK can negatively regulate macropinocytosis-dependent growth. Whether p38 MAPK can regulate lysosomal catabolism in a similar manner as mTOR or whether it acts through an independent mechanism is an interesting question [31]. These further studies will help unravel the underlying mechanisms of the positive and negative regulators of macropinocytosis-dependent growth identified in the current study and help elucidate novel targets for regulating macropinocytosis in cancer cells.

## Data Availability

Data is contained within the article or supplementary material.

## Supplementary Materials

The following supporting information can be downloaded at: https://www.mdpi.com/article/10.3390/metabo12090831/s1, Table S1: Compounds that lead to a decrease or increase in AUC.
